# IRF1 and NF-kB Restore MHC Class I-Restricted Tumor Antigen Processing and Presentation to Cytotoxic T Cells in Aggressive Neuroblastoma

**DOI:** 10.1371/journal.pone.0046928

**Published:** 2012-10-05

**Authors:** Silvia Lorenzi, Matteo Forloni, Loredana Cifaldi, Chiara Antonucci, Arianna Citti, Renata Boldrini, Marco Pezzullo, Aurora Castellano, Vincenzo Russo, Pierre van der Bruggen, Patrizio Giacomini, Franco Locatelli, Doriana Fruci

**Affiliations:** 1 Paediatric Haematology/Oncology Department, Bambino Gesù Children’s Hospital, IRCCS, Rome, Italy; 2 Pathology Department, Bambino Gesù Children’s Hospital, IRCCS, Rome, Italy; 3 Cancer Gene Therapy Unit, Scientific Institute San Raffaele, Milan, Italy; 4 Laboratory of Immunology CRS, Regina Elena National Cancer Institute, Rome, Italy; 5 Ludwig Institute for Cancer Research and Université Catholique de Louvain, de Duve Institute, Brussels, Belgium; 6 University of Pavia, Pavia, Italy; University of Bergen, Norway

## Abstract

Neuroblastoma (NB), the most common solid extracranial cancer of childhood, displays a remarkable low expression of Major Histocompatibility Complex class I (MHC-I) and Antigen Processing Machinery (APM) molecules, including Endoplasmic Reticulum (ER) Aminopeptidases, and poorly presents tumor antigens to Cytotoxic T Lymphocytes (CTL). We have previously shown that this is due to low expression of the transcription factor NF-kB p65. Herein, we show that not only NF-kB p65, but also the Interferon Regulatory Factor 1 (IRF1) and certain APM components are low in a subset of NB cell lines with aggressive features. Whereas single transfection with either IRF1, or NF-kB p65 is ineffective, co-transfection results in strong synergy and substantial reversion of the MHC-I/APM-low phenotype in all NB cell lines tested. Accordingly, linked immunohistochemistry expression patterns between nuclear IRF1 and p65 on the one hand, and MHC-I on the other hand, were observed *in vivo*. Absence and presence of the three molecules neatly segregated between high-grade and low-grade NB, respectively. Finally, APM reconstitution by double IRF1/p65 transfection rendered a NB cell line susceptible to killing by anti MAGE-A3 CTLs, lytic efficiency comparable to those seen upon IFN-γ treatment. This is the first demonstration that a complex immune escape phenotype can be rescued by reconstitution of a limited number of master regulatory genes. These findings provide molecular insight into defective MHC-I expression in NB cells and provide the rational for T cell-based immunotherapy in NB variants refractory to conventional therapy.

## Introduction

Neuroblastoma (NB) is a childhood solid tumor derived from precursor cells of the sympathetic nervous system which accounts for 15% of pediatric cancer deaths [1]. Clinical features of the tumor are very heterogeneous, ranging from spontaneous regression to rapid progression. The latter has been shown to be associated with several genomic and functional abnormalities, including *MYCN* gene amplification and overexpression, allelic loss of chromosome 1p, 3p, 11q and 14q, gains of 1q and 17q, as well as deficient MHC class I (MHC-I) expression and antigen presentation [2]–[6].

Despite intensive multimodal treatment (chemotherapy, surgery, external beam radiation therapy, myeloablative chemotherapy with stem cell rescue, differentiating therapy with cis-retinoic acid and immunotherapy), clinical outcome of high-risk NB remains poor, with less than 30% long-term remissions [7]. Thus, novel alternative therapeutic approaches are desirable to improve survival. T cell-based immunotherapy is an attractive option for treatment of chemotherapy-refractory/high-risk NB patients, since NB expresses known T-cell tumor antigens from the MAGE family, GAGE, NY-ESO-1, PRAME, tyrosine hydroxylase, survivin, MYCN, and Anaplastic Lymphoma Kinase (ALK) [8]–[20]. However, NB poorly presents these antigens due to a complex, coordinated low expression of MHC-I and many gene products collaborating to build functional MHC-I molecules and their antigen cargo [21]–[23]. These include β2-microglobulin (β2m), the Transporter Associated with Antigen Processing (TAP1 and TAP2) subunits, the Endoplasmic Reticulum Aminopeptidases (ERAP) 1 and ERAP2, and the peptide antigen editor tapasin (TPN) [24]. Low expression of these genes, often collectively referred to as members of the antigen processing machinery (APM), drastically limits the applicability of T-cell immunotherapy in NB.

IFN-γ has been demonstrated to enhance and reconstitute the MHC-I antigen processing and presentation pathway [25]. Unfortunately, the immunotherapeutic use of IFNs in human solid tumors has met limited success [26], [27]. In fact, IFN-γ induces negative feedback loops preventing long-lasting biological effects [28], [29]. Adverse reactions and negative impact on tumor progression and clinical outcome of the disease have been reported upon systemic administration of this agent in clinical trials [26]. In view of these considerations, it would be desirable to identify which alternative factors are necessary and sufficient to elicit full antigen presentation in NB, without incurring the complication of *in vivo* treatment with a pleiotropic lymphokine.

IFN-γ and TNF-α induce a number of MHC-I transcriptional activators including the interferon regulatory factors (IRF) 1 and IRF2 and the transcription factor NF-kB, respectively [30]–[32]. These, in turn, bind to adjacent *cis* regulatory sequences shared by MHC-I and APM molecules, such as the enhancer A element and ISRE-like sequences, respectively [33]–[39]. Recently, we have shown that NF-kB is the major, direct transacting factor responsible for coordinated regulation of MHC-I and certain APM components in NB, and that reconstitution of this missing transacting function enhances MHC-I in at least some aggressive NB cell lines from stroma-poor lesions [38].

Herein, we demonstrate that wide-range APM reconstitution requires proper selection of additional master MHC-I regulators. Specifically, IRF1 synergizes with NF-kB in reconstituting full MHC-I-restricted tumor antigen processing and antigen presentation to cytotoxic T-cell (CTL) clones. These findings pinpoint a crucial node of immunotherapeutic intervention on NB.

## Materials and Methods

### Tumor Cell Lines and Reagents

All human NB and melanoma cell lines were obtained from the American Type Culture Collection and characterized by morphology and HLA class I typing by PCR-SSP sets (Genovision). Cells were grown in different media: ET1 and MSR3-mel cells were maintained in IMDM, SH-SY5Y cells and SK-N-SH cells in DMEM and MEM, respectively, and the other cell lines were maintained in RPMI 1640 medium. All media were supplemented with 10% FCS (HyClone), glutamine, 100 µg/ml penicillin and 50 µg/ml streptomycin. For IFN-γ-treatment, NB cell lines were cultured for 48 hours in the presence of 500 U/ml recombinant human IFN-γ (R & D Systems).

### DNA Constructs and Transfections

IRF1, IRF2 and NF-kB p65 (kindly provided by A. Battistini, Department of Infectious, Parasitic and Immune-Mediated Diseases, Istituto Superiore di Sanità, Rome, Italy and M. Levrero, Rome Oncogenomic Center, Regina Elena Institute, Rome, Italy, respectively) and the corresponding empty vectors were transfected using Lipofectamine 2000 (Invitrogen). Cells were seeded in tissue culture plates and transfected 24 hours later at an 80% confluence with DNA-lipofectamine complexes in OptiMEM. Fifteen hours following transfection the culture medium was replaced with fresh medium. For IRF1 and IRF2 silencing, cells (40% confluent) were transfected with 80 nmol/L siRNAs or scrambled siRNA using Lipofectamine 2000. Cell were collected 40 hours after transfection.

### Reverse Transcription and Real-time Quantitative PCR

Total RNA was extracted with Trizol Reagent (Invitrogen) and retro-transcribed with SuperScript II (Invitrogen). RT-PCR and real-time PCR analyses were performed as previously described [38], [40]. 18S RNA and GAPDH were used for normalization.

### Antibodies, Immunoblotting and Flow Cytomety

Murine mAbs W6/32, HC10, 6H9, 3F5, NAMB-1 and 435.3 recognize human fully assembled MHC-I heavy chains, β2m-free MHC-I heavy chains, ERAP1, ERAP2, β2m and TAP2, respectively [41]–[45]. The rabbit polyclonal Abs R5996-4, 352 and GCY were raised against human β2m-free MHC-I heavy chains, tapasin and TAP1, respectively [38], [41]. The following Abs were all from Santa Cruz Biotechnology: NF-kB p65 (sc-109), IRF1 (sc-497), IRF2 (sc-498) and PCNA (sc-56). The polyclonal Ab IRF1 (Ab-26109) from Abcam was used for immunohistochemical staining. Whole-cell and nuclear extracts obtained as previously described [38], were quantified by the bicinchoninic acid assay (Pierce), resolved on 8–15% SDS-PAGE and electroblotted. Filters were probed with primary Abs followed by incubation with peroxidase-coupled secondary Ab. ERp57 and PCNA were used as loading control of whole-cell and nuclear extracts, respectively. MHC-I surface expression was determined by flow cytometry with mAb W6/32 on a FACSCalibur (Becton Dickinson).

### Patients and Immunohistochemistry

All tumor specimens were obtained from NB patients diagnosed at the “Bambino Gesù” Children Hospital, Rome, (Italy), after obtaining written informed parental consent and approval by the Ethical Committee of the Institution. The histologic features of NB were classified into ganglioneuroblastoma (stroma rich, 10 cases) and undifferentiated NB (stroma poor, 10 cases) according to the percentage and degree of differentiation of the NB cells using the criteria of the International NB Pathology Classification [46]. For immunohistochemical staining, consecutive sections of paraffin-embedded tissue blocks were cut at 3 µm. Deparaffinization and antigen retrieval were performed with PT-link (Dako) in Tris/EDTA (pH 9.0) (Dako) for 15 min at 98°C for HC10 and citrate buffer (pH 6.1) (Dako) for the other Abs. Sections were incubated with the primary mAb for 45 min at room temperature, followed by incubation with the peroxidase-conjugated secondary Ab (Dako) for 20 min at room temperature. Diaminobenzidine-tetrahydrochloride (DAB) was used as chromogen. All samples were counterstained with hematoxylin.

### Antigen-specific T Cells Generation and Propagation

Peripheral blood mononuclear cells (PBMC) from an HLA-A1 melanoma patient were incubated with the MAGE-A3.A1 peptide EVDPIGHLY (20 mM) [47] for 60 min at room temperature. Then, cells were washed and cultured in complete medium containing 10% human serum, rhIL-2 (20 U/ml) and rhIL-7 (10 U/ml) [48]. After 10 days, the cells were stimulated weekly with autologous MAGE-A3.A1 peptide-pulsed dendritic cells (DCs) in the presence of irradiated (100 Gy) autologous EBV transformed B-cells, in complete medium containing 10% human serum and rhIL-2 (50 U/mL) [48], [49]. Generation of CTLs specific to the MAGE-A3.A1 peptide was verified by a standard 5-hour ^51^Cr release assay and an IFN-γ ELISA assay against the MAGE-A3^+^ HLA-A1^+^ melanoma cell line ET-1, MAGE-A3^+^ HLA-A1^−^ melanoma cell line 624mel and the HLA class I negative melanoma cell line MSR-3-mel as controls. The presence of natural killer (NK) cell activity was tested by cold inhibition assay using the NK targets K562 cells. Lytic activity of the CTLs against NB cells was evaluated by ^51^Cr release assay.

**Figure 1:**
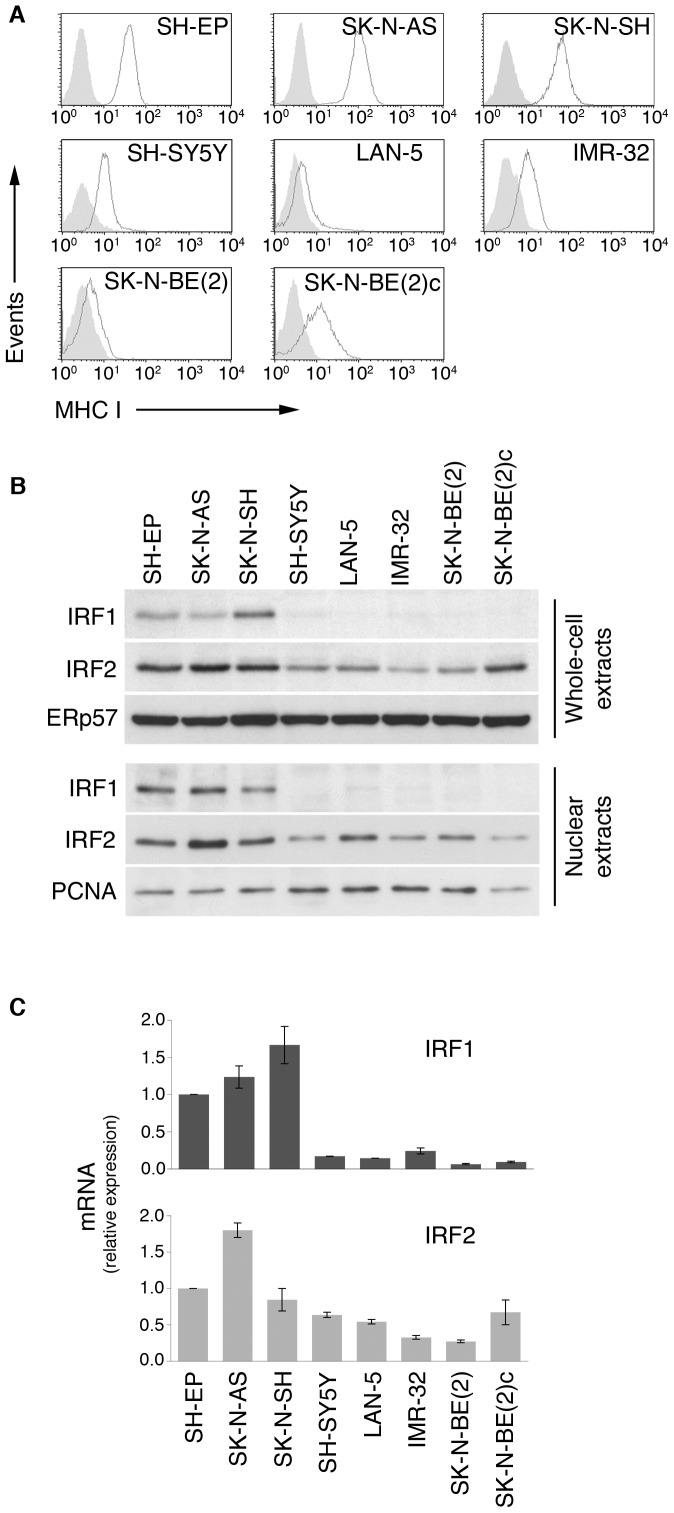
Expression of MHC-I, IRF1 and IRF2 in NB cell lines. A, flow cytometry analysis of surface MHC-I expression in NB cell lines using W6/32 mAb (grey lines). Shaded histograms, negative controls stained with isotype-matched primary antibody. B, immunoblot analysis of IRF1 and IRF2 in NB cell lines. Equal amounts of whole-cell extracts and nuclear extracts, as indicated, were resolved by SDS-PAGE, immunoblotted and probed with specific antibodies. ERp57 and PCNA were used for normalization. Positive and negative IRF1 and IRF2 controls, as well as densitometric and statistical analysis of WB bands are shown in Fig. S1. C, qRT-PCR analysis of mRNAs from different NB cell lines. 18S RNA was used for normalization. Significant differences between the 3 MHC-I-expressing NB cells and the 5 MHC-I-low NB cells of mRNA expression separately averaged were evaluated as described in Materials and Methods. IRF1: *P*<0,001; IRF2: *P* = 0,09; ± SD of triplicate assays. Data shown in the panels A to C are representative of at least 3 independent experiments.

### Statistical Analysis

Digital images of Western blots were analysed by Image J (http://rsbweb.nih.gov/ij/index.html) and statistical significance of densitometric values was assessed by the two-tailed unpaired Student’s *t*-test. A P value of <0.05 was considered to be statistically significant.

## Results

### IRF1 and IRF2 Expression Correlates with MHC-I Expression in NB Cell Lines and Primary NB Samples

Flow cytometry and Western blotting of NB cell lines revealed that the 3 cell lines expressing higher levels of cell surface MHC-I (SH-EP, SK-N-AS and SK-N-SH) also expressed higher levels of IRF1 and IRF2, whereas the 5 cell lines expressing lower levels of cell surface MHC-I displayed lower levels of IRF1 and IRF2 (Figs. 1A, 1B and S1). Similar results were obtained in nuclear extracts (Fig. 1B and S1) and at mRNA level (Fig. 1C).

**Figure 2:**
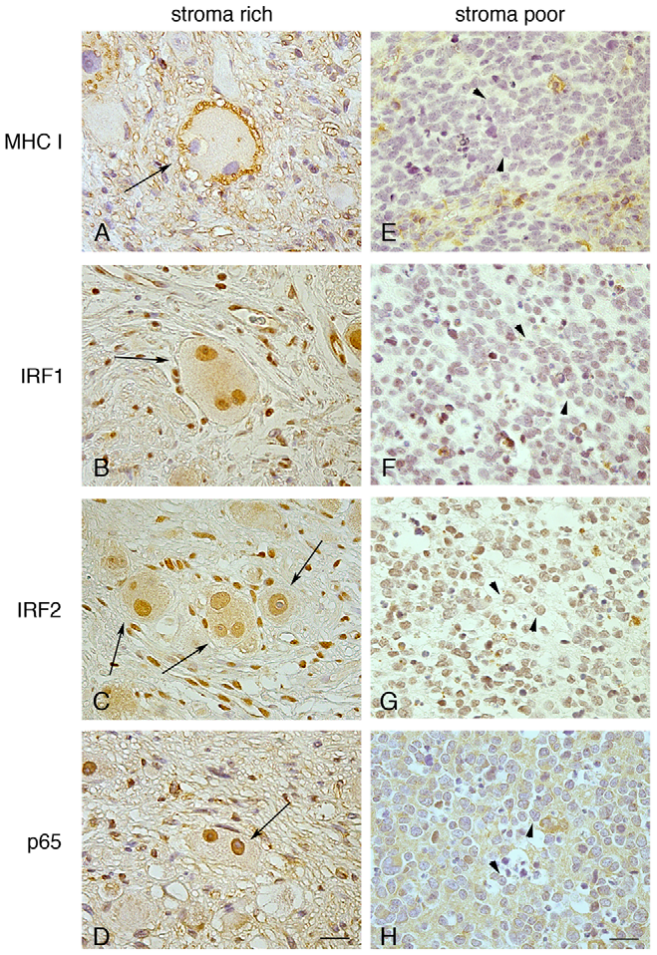
Expression of MHC-I, IRF1, IRF2 and the NF-kB p65 subunit in primary NB lesions. Immunohistochemistry of human NB tissue sections with Abs to MHC-I (A, E), IRF1 (B, F), IRF2 (C, G) or NF-kB p65 subunit (D, H). Visualized with diaminobenzidine (DAB; brown), nuclei counter-stained with haematoxilin (blue). IRF1, IRF2 and NF-kB p65 are strongly expressed in the nuclei of mature ganglion cells (arrows), endothelial cells, lymphocytes and stroma cells in the well-differentiated MHC-I-positive ganglioneuroblastoma (A-D), and weakly expressed in the MHC-I-negative neuroblastic cells (arrowhead), i.e. undifferentiated stroma-poor NB (E-H). In E-H, positive staining of benign cells, including lymphocytes and macrophages. NF-kB p65-positive staining of the fibrillary network in H is evident. Original magnification, x40. Scale bars 30 µm. Data shown are representative of 10 stroma-rich and 10 stroma-poor NB tissue sections.

IRF1, IRF2, and the NF-kB p65 subunit were clearly detected by immunohistochemistry in the nuclei of MHC-I-positive, mature ganglion cells, in 10/10 primary stroma-rich NB lesions. Conversely, the three transcription factors were hardly seen in the nuclei of MHC-I-negative neuroblastic cells from 10/10 stroma-poor (undifferentiated) NB samples. Representative staining (Figs. 2 and S2) reveals expression levels and subcellular localization. Thus, a significant correlation was detected, both *in vitro* and *in vivo*, between MHC-I, NF-kB, IRF1 and IRF2.

### IFN-γ Enhances the Expression of IRFs, MHC-I and APM Components in NB Cell Lines

**Figure 3:**
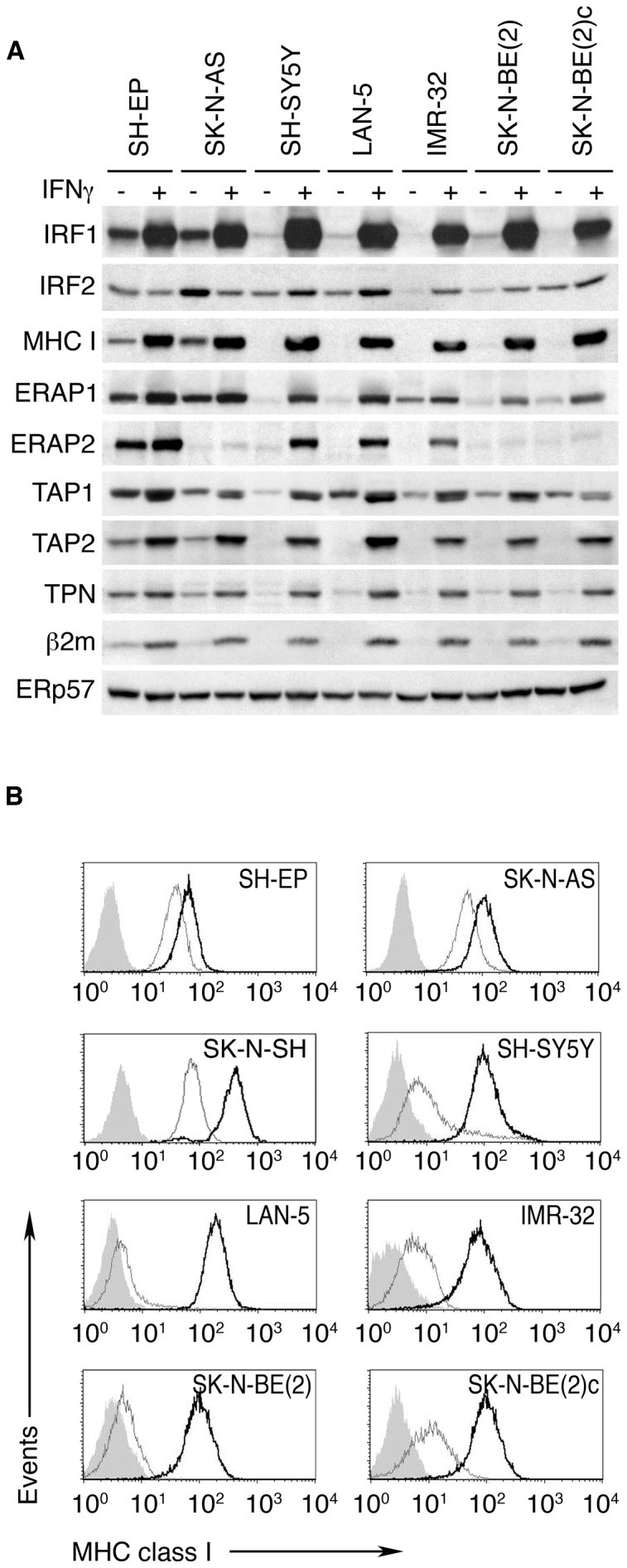
IFN-γ restores IRF1, MHC-I and APM components in NB cell lines. A, whole-cell lysates of NB cell lines cultured for 48 hours in the presence and absence of IFN-γ were resolved by SDS-PAGE and Western blotted with specific antibodies. ERp57 was used as loading control. Densitometric and statistical analysis of WB bands are shown in Fig. S4. B, flow cytometry analysis with mAb W6/32 of surface MHC-I expression of NB cells grown, as above, for 48 hours in the presence and absence of IFN-γ. Untreated cells (grey lines), treated cells (black lines) and isotype-matched negative controls (shaded histograms) are shown. Data shown in panels A and B are representative of 3 and 5 independent experiments, respectively.

NB cell lines were treated with IFN-γ, a major enhancer of MHC-I, APM components, IRF1 and IRF2. Western blot analysis revealed coordinated expression and significant enhancement of IRF1, MHC-I and almost all the APM gene products tested in all the cells, although the magnitude of the enhancement tended to be greater in the 5 cell lines expressing lower baseline levels, grouped at the right-hand side of the panel (Fig. 3A, S3 and S4). These cells also displayed preferential up-regulation of IRF2, a known mediator of IFN-γ signaling extinction that competitively inhibits the binding of IRF1 to the ISRE (Fig. 3A, S3 and S4). Consistent with polypeptide enhancement, cell surface MHC-I expression was strongly increased in all NB cell lines (Fig. 3B).

Thus, constitutive and IFN-γ-mediated IRF1/IRF2 expression correlates with constitutive and IFN-γ-mediated MHC-I and APM expression, suggesting that IRFs are involved in regulating MHC-I in NB.

### Synergistic Enhancement of MHC-I and APM Component Expression by IRF1 and NF-kB in NB Cell Lines

**Figure 4:**
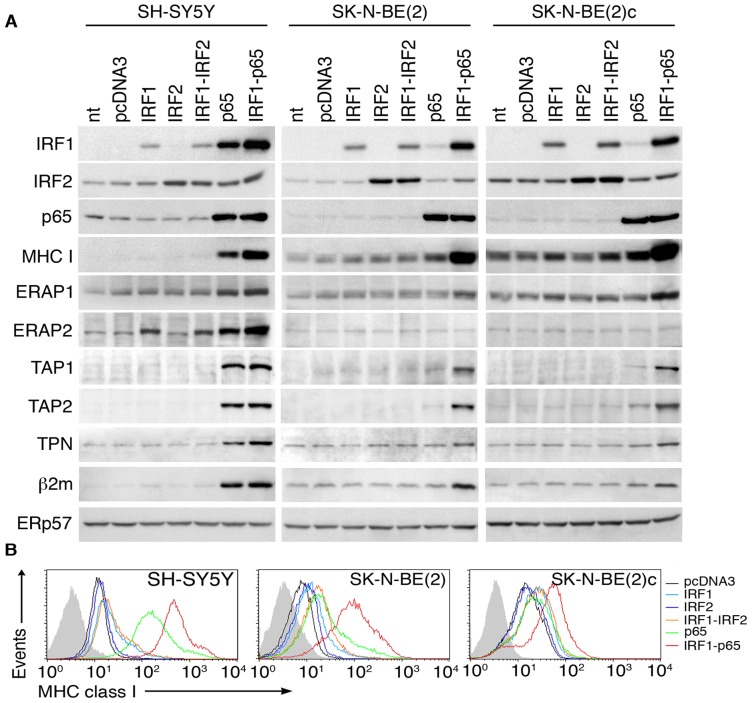
IRF1 and p65 synergistically enhance MHC-I and APM components in NB cell lines. A, immunoblotting of cell extracts from three NB cell lines left untransfected (none) or single- and double-transfected, as indicated, with the control empty vector (pcDNA3) or vectors expressing IRF1, IRF2, and NF-kB p65. ERp57 was used as loading control. Densitometric and statistical analysis of WB bands related to the cells transfected with pcDNA3, or p65, or IRF1 and p65 are shown in Fig. S5. B, flow cytometry analysis of surface MHC-I expression in the same NB cell lines (indicated by different colors) with mAb W6/32. Isotype-matched negative controls are displayed as shaded histograms. Data shown in panels A and B are representative of 5 and 8 independent experiments, respectively.

Combined with our previous study [38], the above observations strongly indicate that not only a lack of NF-kB, but also a lack of IRF1 and/or IRF2, is responsible for the MHC-I-low NB phenotype. To obtain direct evidence for this, the three NB cell lines SH-SY5Y, SK-N-BE(2) and SK-N-BE(2)c, which are particularly depressed in MHC-I expression, were single-transfected and double-transfected with expression vectors encoding IRF1, IRF2, and the NF-kB p65 cDNAs (Fig. 4A). IMR-32 and LAN-5 could not be included in this analysis because they were refractory to transfection.

Transfection-mediated overexpression of either IRF1 or IRF2 did not affect NF-kB p65, MHC-I or APM components in any of the three tested cell lines, the only exceptions being IRF1-mediated up-regulation of ERAP2 and MHC-I in SH-SY5Y and SK-N-BE(2)c, respectively (Fig. 4A). No additional up-regulation could be obtained by co-transfection of IRF1 and IRF2, demonstrating that the IRFs, by themselves, are very poor MHC-I/APM transactivators in NB cell lines. In line with the known antigen-presentation impairment operating in NB, even the master MHC-I regulator NF-kB p65 was partially effective, since it up-regulated both MHC-I and APM components in SH-SY5Y, but only MHC-I in SK-N-BE(2) and SK-N-BE(2)c (Figs. 4A and S5). Remarkably, these two cell lines are MYCN amplified and representative of very aggressive NB tumors [38]. Also noteworthy is the observation that only in fully responsive SH-SY5Y cells was p65 capable of promoting substantial IRF1 enhancement, suggesting that efficient correction of APM-low phenotypes requires not only a direct effect of p65 on target genes, but also an indirect effect through IRF1 (Figs. 4A and S5). In agreement with this interpretation, nearly complete MHC-I/APM reconstitution could be obtained in refractory SK-N-BE(2) and SK-N-BE(2)c cells through double IRF1/p65 transfection, ERAP2 remaining the only unresponsive gene (Figs. 4A and S5). This is reminiscent of experiments in figure 3, in which IFN-γ also failed to up-regulate ERAP2 in SK-N-BE(2) and SK-N-BE(2)c, and suggests a gene-specific up-regulation defect in these cell lines.

**Figure 5:**
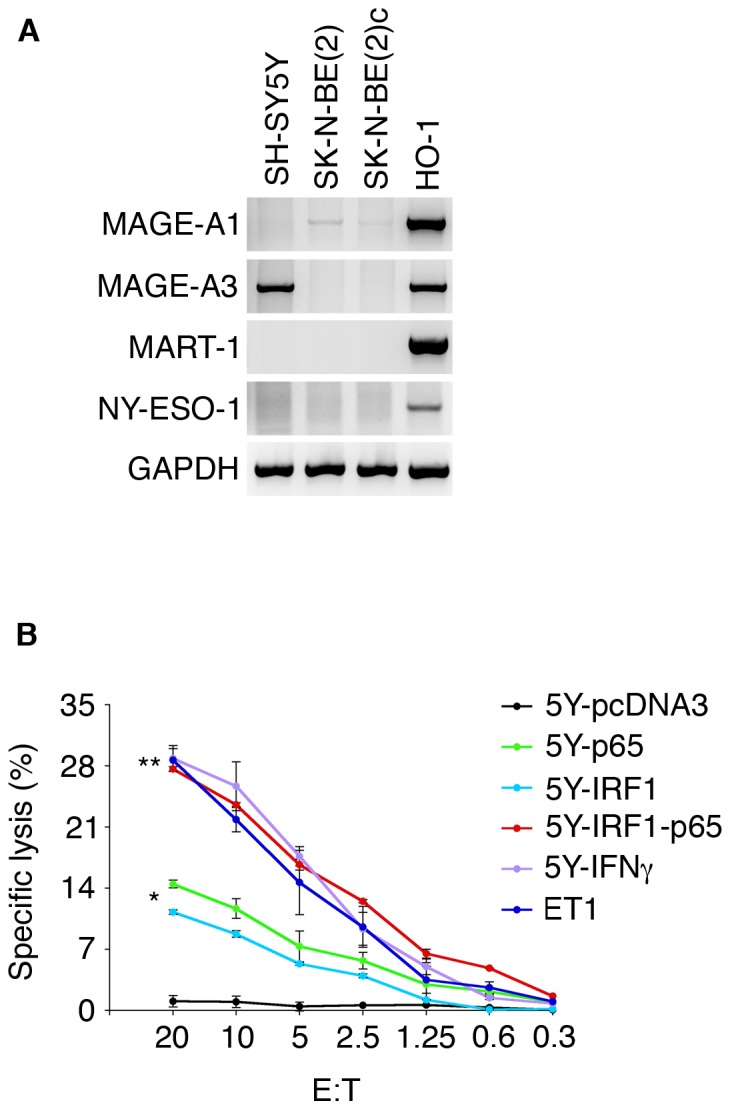
Double IRF1/p65 transfection renders NB cells susceptible to killing by specific cytotoxic T cells. A, RT-PCR analysis of tumor antigens in NB cell lines. The melanoma cell line HO-1 was included as positive control. GAPDH gene expression was used for normalization. B, the SH-SY5Y cell line (5Y) grown in the presence of IFN-γ (5Y-IFN-γ) for 48 hours or co-transfected with IRF1 (5Y-IRF1) and the NF-kB p65 subunit (5Y-IRF1-p65, and 5Y-p65 respectively) or empty vector (5Y-pcDNA3) was assayed by either MHC-I expression (Fig. S6B) by flow cytometry or as targets to HLA-A1-restricted/MAGE-A3-specific CTLs at the indicated effector:target (E:T) ratios in a standard ^51^Cr-release assay. Statistically significant differences are indicated (*, P<0.002, **, P<0.0000002). Mean ± SD of three experiments is shown. Data shown in A and B are representative of 3 experiments.

In agreement with the above experiments, optimal recovery of cell surface MHC-I expression could only be observed in double p65/IRF1 transfectants (Fig. 4B), conclusively showing critical dependence of MHC-I/APM reactivation on NF-kB/IRF1 synergy, particularly in certain NB cells with hard-to-rescue APM defects.

### Up-regulation of Cell Surface MHC-I Expression with IRF1 and NF-kB p65 Renders NB Cells Susceptible to Antigen-specific CTLs

NB cells are not susceptible to tumor antigen-specific CTLs because of their very low MHC-I expression [23]. Hence, we sought to determine whether an increase in cell surface MHC-I expression by IRF1 and p65 would render NB cells susceptible to antigen-specific T-cell cytotoxicity.

To this end, the three NB cell lines tested above, i.e. SH-SY5Y, SK-N-BE(2) and SK-N-BE(2)c, were evaluated by RT-PCR for the expression of the well-known tumor-antigen coding genes *MAGE-A1*, *MAGE-A3*, *MART-1* and *NY-ESO-1*. Three antigens (*MAGE-A1*, *MART-1* and *NY-ESO-1*) were not detectable. Absence of MART-1 in NB is not surprising in light of a previous study [8]. Only *MAGE-A3* was highly expressed in the HLA-A1^+^ SH-SY5Y cell line (Fig. 5A). Therefore, SH-SY5Y cells treated with IFN-γ, or transfected with IRF1 and/or p65 were tested in a standard ^51^Cr release assay as targets of HLA-A1-restricted CTL specific for MAGE-A3-encoded peptide EVDPIGHLY [48]. CTLs did not lyse the HLA class I-negative melanoma cell line MSR3-mel (not shown) and SH-SY5Y cells transfected with an empty vector, but lysed efficiently, and approximately to the same extent, the MAGE-A3^+^ HLA-A1^+^ melanoma cell line ET-1, SH-SY5Y cells treated with IFN-γ, and SH-SY5Y cells co-transfected with IRF1 and p65 (Figs. 5B and S6B). Conversely, SH-SY5Y single-transfected with IRF1 or p65 were lysed at lower efficiency as compared to SH-SY5Y double-transfected cells (Figs. 5B and S6B). These lytic effects are entirely due to MHC-I rescue, since IRF1 and/or p65 transfections did not alter the expression of MAGE-A3 (S6A).

Altogether, these data provide the proof of principle that the efficacy of T cell-based immunotherapy of NB may be improved by enhancing cell surface MHC-I expression with IRF1 and NF-kB p65.

## Discussion

NB is the human tumor in which MHC-I-low phenotypes have the highest prevalence and most closely correlate with molecular determinants of aggressiveness [4]. In this report, we used NB cell lines representative of distinct NB molecular classes to identify impaired steps of MHC-I antigen presentation, and to correct them.

We confirm that NF-kB is the master regulator of constitutive MHC-I expression in NB, but, in addition, we show that it is a poor inducer of some APM components in NB cells with aggressive features and low MHC-I expression. However, these cells are particular susceptible to IFN-γ up-regulation as demonstrated by the elective IFN-γ-mediated MHC-I up-regulation and by the induction of both IRF1 and its inhibitor IRF2. These results suggest a full-fledged activation-extinction signaling loop. Accordingly, IRF1 is absolutely necessary to obtain a full rescue of antigen-presenting molecules in these MHC-I-low cells.

MHC-I reconstitution requires synergy between IRF1 and NF-kB, since IRF1 (either alone or in combination with IRF2) is substantially inactive. This finding places IRF1 on a hierarchically lower position than NF-kB. Nevertheless, IRF1 also appears to be crucial. Without it, large sections of the antigen processing and presentation cascade are impaired.

IRF1 has long been known to be a major IFN-γ-induced mediator of MHC-I up-regulation in tumors, including NB [21]. However, a direct effect on MHC-I and APM has never been demonstrated, to our knowledge, by direct reconstitution with IRF1. Herein, we identify IRF1 as a crucial coordinator of APM up-regulation. In our hands, IRF1 and NF-kB synergize in enhancing MHC-I, β2m, TAP1, TAP2, ERAP1, ERAP2, and tapasin, which are structural MHC-I subunits, dedicated peptide translocators, trimmers, editors, and chaperones.

Along the same line, IRF1, NF-kB p65, and MHC-I are coordinately expressed *in vivo* in tumor lesions, their nuclear absence and presence neatly segregating between high-grade and low-grade NB, respectively. Altogether, these findings suggest that linked suppression of genes collaborating in antigen processing and presentation in aggressive NB depends, *in vivo* as well as *in vitro*, on epistatic repression mainly acting on IRF-1 and NF-kB. Downstream correction of the stoichiometric insufficiency of IRF1 and NF-kB restores the presentation of tumor antigens in a model NB cell line, providing the proof of principle that complex immune escape phenotypes can be rescued by a limited number of master genes, and that NB can be made sensitive to CTLs that otherwise would be unable to recognize expressed, endogenous NB tumor antigens. To our knowledge, this is the first complete reconstitution of the MHC-I antigen-processing pathway in a tumor model by reintegration of a limited number of transcription factors, and not through treatment with pleiotropic cytokines or a mixture of undefined constituents. It is remarkable that at least in some NB cell lines MHC-I/APM defects are fully reversible, and that, in the limited cell panel studied by us, NF-kB and IRF1 are as good as IFN-γ in rescuing low expression.

Our inability to correct ERAP2-low phenotypes in certain cells also suggests that extending the panel of NB cell lines and tissues is likely to reveal additional requirements. Further studies are necessary to test the percentage of NB tumors that can be rescued by double NF-kB/IRF1 reconstitution. It will be of interest to identify the master regulators that are still missing to achieve step-by-step, full reconstitution of the antigen presentation pathway in as many NB tumors as possible.

ERAP1 and ERAP2 trim peptide antigens to a size that fits the MHC-I antigen-binding groove. Whereas interference with the mouse homologue of ERAP1 has been shown to drastically alter tumor recognition by both T and NK cells [50], ERAP2 has no known mouse homologue, is less tightly coordinated with MHC-I [51], [52] *in vitro* and *in vivo*, and is believed to have a more specialized role than ERAP1, i.e. ERAP2 only trims a limited subset of peptide antigens. Therefore, whereas of interest in light of the specialized role and regulation of ERAP2, the selective non-rescuability of this aminopeptidase in some NB cell lines is not expected to affect the processing of most antigens, including tumor antigens.

The present results support the original suggestion that MHC-I and APM components act as a coordinome with core and peripheral (gene-specific) control features, and a hierarchy of control steps [41]. Rescue of tumor antigen presentation on the whole by a limited number of transactivators will hopefully provide impetus to search for NB antigens to be used as immunotherapeutic targets, and to design small molecules to selectively manipulate the expression of NF-kB and IRF1 transcription factors, and target their activity to tumor cells. Indeed, antineoplastic effects of small molecule inhibitors targeting a single transcription factor (the late SV40 factor) have been preliminary reported [53]. It is hoped that antigen-specific immunotherapy (altogether one of the safest therapeutic approaches in oncology) may be more successful in early childhood, in the unexplored context of the very plastic immune system of the infant.

## Supporting Information

Figure S1 **Specificity, densitometric and statistical analyses of IRF1 and IRF2 expression.** (A) Immunoblot analysis of IRF1 and IRF2 in SH-EP cells transfected with either IRF1 siRNA (siIRF1), IRF2 siRNA (siIRF2) or control scrambles (scr). The Jurkat cell line was included as a positive control. Equal amounts of whole-cell extracts were resolved by SDS-PAGE, immunoblotted and probed with IRF1 and IRF2 antibodies. ERp57 was used for normalization. Silencing of IRF1 and IRF2 by RNA interference in SH-EP cells affects the levels of IRF1 and IRF2 polypeptides as compared with the controls demonstrating the specificity of the antibodies. (B) Densitometric and statistical analyses of the Western blot bands shown in Figure 1B. Western blot bands in Fig. 1B were submitted to densitometric analysis. IRF1 and IRF2 values of the 3 MHC-I-expressing NB cells (to the left) and the 5 MHC-I-low NB cells (to the right) were separately averaged, and statistical significance (*P* value) of the differences between the two series was calculated as described in Materials and Methods.
(TIF)

Figure S2 **Immunohistochemistry of human NB tissue sections with antibodies to MHC-I (A, F), IRF1 (B, G), IRF2 (C, H), p65 (D, L) or negative controls (E, L) at original magnification, x20.** Scale bars 60 µm. Expression of IRF1, IRF2 and p65 in the nuclei of mature ganglion cells (arrows), endothelial cells, lymphocytes and stroma cells in the MHC-I-positive ganglioneuroblastoma is shown in A to D. Weak expression of IRF1, IRF2 and p65 in the MHC-I-negative neuroblastic cells (arrowhead), is shown in F to I. Data shown are representative of 10 stroma-rich and 10 stroma-poor NB tissue sections.
(TIF)

Figure S3 **Lysates of SK-N-SH and SH-SY-5Y cell lines cultured for 48 hours in the presence and absence of IFN-γ were resolved by SDS-PAGE and immunoblotted and probed with the indicated antibodies.** ERp57 was used for normalization. Data are representative of 3 independent experiments.
(TIF)

Figure S4 **Densitometric and statistical analyses of the immunoblots shown in** **Figure 3A** **.** Densitometric values of the IFN-γ-treated (+IFNγ) or left untreated NB cells were separately averaged, and statistical significance (*P* value) of the differences between the two series was calculated as described in Materials and Methods. In addition, densitometric values of the 3 MHC-I-expressing NB cells and the 5 untreated MHC-I-low NB cells both untreated were separately averaged, and statistical significance (*P* value) of the differences between the two series was calculated as described above.
(TIF)

Figure S5 **Densitometric and statistical analyses of the immunoblots shown in** **Figure 4A** **.** Western blot bands were submitted to densitometric analysis. Densitometric values of cells transfected with pcDNA3, NF-kB p65 subunit, or IRF1 and NF-kB p65 subunit were separately averaged, and statistical significance (*P* value) of the differences between the three series was calculated as described in Materials and Methods.
(TIF)

Figure S6 A, RT-PCR analysis of MAGE-A3 in the SH-SY5Y either untransfected (none) and transfected with IRF1 and/or the NF-kB p65 subunit and the control empty vector (pcDNA3). Total mRNA was extracted from the transfected cells, reverse transcribed and cDNAs amplified with specific primers for MAGE-A3. GAPDH gene was used for normalization. B, flow cytometry analysis of surface MHC-I expression of SH-SY5Y cells treated as described in Figure 5 using W6/32 mAb. Data are shown as mean of fluorescence.
(TIF)

## References

[1] Maris JM (2010) Recent advances in neuroblastoma. N Engl J Med 362: 2202–2211. 20558371 10.1056/NEJMra0804577 PMC3306838

[2] Schwab M, Varmus HE, Bishop JM, Grzeschik KH, Naylor SL, et al (1984) Chromosome localization in normal human cells and neuroblastomas of a gene related to c-myc. Nature 308: 288–291. 6700732 10.1038/308288a0

[3] Seeger RC, Brodeur GM, Sather H, Dalton A, Siegel SE, et al (1985) Association of multiple copies of the N-myc oncogene with rapid progression of neuroblastomas. N Engl J Med 313: 1111–1116. 4047115 10.1056/NEJM198510313131802

[4] Feltner DE, Cooper M, Weber J, Israel MA, Thiele CJ (1989) Expression of class I histocompatibility antigens in neuroectodermal tumors is independent of the expression of a transfected neuroblastoma myc gene. J Immunol 143: 4292–4299. 2687378

[5] Attiyeh EF, London WB, Mosse YP, Wang Q, Winter C, et al (2005) Chromosome 1p and 11q deletions and outcome in neuroblastoma. N Engl J Med 353: 2243–2253. 16306521 10.1056/NEJMoa052399

[6] Drew PD, Lonergan M, Goldstein ME, Lampson LA, Ozato K, et al (1993) Regulation of MHC class I and beta 2-microglobulin gene expression in human neuronal cells. Factor binding to conserved cis-acting regulatory sequences correlates with expression of the genes. J Immunol 150: 3300–3310. 8468472

[7] Matthay KK, O’Leary MC, Ramsay NK, Villablanca J, Reynolds CP, et al (1995) Role of myeloablative therapy in improved outcome for high risk neuroblastoma: review of recent Children’s Cancer Group results. Eur J Cancer 31A: 572–575. 7576971 10.1016/0959-8049(95)00015-b

[8] Corrias MV, Scaruffi P, Occhino M, De Bernardi B, Tonini GP, et al (1996) Expression of MAGE-1, MAGE-3 and MART-1 genes in neuroblastoma. Int J Cancer 69: 403–407. 8900375 10.1002/(SICI)1097-0215(19961021)69:5<403::AID-IJC9>3.0.CO;2-9

[9] Wolfl M, Jungbluth AA, Garrido F, Cabrera T, Meyen-Southard S, et al (2005) Expression of MHC class I, MHC class II, and cancer germline antigens in neuroblastoma. Cancer Immunol Immunother 54: 400–406. 15449039 10.1007/s00262-004-0603-z PMC11034322

[10] Jacobs JF, Brasseur F, Hulsbergen-van de Kaa CA, van de Rakt MW, Figdor CG, et al (2007) Cancer-germline gene expression in pediatric solid tumors using quantitative real-time PCR. Int J Cancer 120: 67–74. 17019710 10.1002/ijc.22118

[11] Grau E, Oltra S, Martinez F, Orellana C, Canete A, et al (2009) MAGE-A1 expression is associated with good prognosis in neuroblastoma tumors. J Cancer Res Clin Oncol 135: 523–531. 18820946 10.1007/s00432-008-0484-1 PMC12160144

[12] Cheung IY, Cheung NK (1997) Molecular detection of GAGE expression in peripheral blood and bone marrow: utility as a tumor marker for neuroblastoma. Clin Cancer Res 3: 821–826. 9815755

[13] Rodolfo M, Luksch R, Stockert E, Chen YT, Collini P, et al (2003) Antigen-specific immunity in neuroblastoma patients: antibody and T-cell recognition of NY-ESO-1 tumor antigen. Cancer Res 63: 6948–6955. 14583496

[14] Rimoldi D, Romero P, Carrel S (1993) The human melanoma antigen-encoding gene, MAGE-1, is expressed by other tumour cells of neuroectodermal origin such as glioblastomas and neuroblastomas. Int J Cancer 54: 527–528. 8509230 10.1002/ijc.2910540329

[15] Soling A, Schurr P, Berthold F (1999) Expression and clinical relevance of NY-ESO-1, MAGE-1 and MAGE-3 in neuroblastoma. Anticancer Res 19: 2205–2209. 10472332

[16] Oberthuer A, Hero B, Spitz R, Berthold F, Fischer M (2004) The tumor-associated antigen PRAME is universally expressed in high-stage neuroblastoma and associated with poor outcome. Clin Cancer Res 10: 4307–4313. 15240516 10.1158/1078-0432.CCR-03-0813

[17] Lode HN, Huebener N, Zeng Y, Fest S, Weixler S, et al (2004) DNA minigene vaccination for adjuvant neuroblastoma therapy. Ann N Y Acad Sci 1028: 113–121. 15650237 10.1196/annals.1322.012

[18] Coughlin CM, Fleming MD, Carroll RG, Pawel BR, Hogarty MD, et al (2006) Immunosurveillance and survivin-specific T-cell immunity in children with high-risk neuroblastoma. J Clin Oncol 24: 5725–5734. 17179106 10.1200/JCO.2005.05.3314

[19] Himoudi N, Yan M, Papanastasiou A, Anderson J (2008) MYCN as a target for cancer immunotherapy. Cancer Immunol Immunother 57: 693–700. 18004567 10.1007/s00262-007-0409-x PMC11030590

[20] Osajima-Hakomori Y, Miyake I, Ohira M, Nakagawara A, Nakagawa A, et al (2005) Biological role of anaplastic lymphoma kinase in neuroblastoma. Am J Pathol 167: 213–222. 15972965 10.1016/S0002-9440(10)62966-5 PMC1603453

[21] Corrias MV, Occhino M, Croce M, De Ambrosis A, Pistillo MP, et al (2001) Lack of HLA-class I antigens in human neuroblastoma cells: analysis of its relationship to TAP and tapasin expression. Tissue Antigens 57: 110–117. 11260505 10.1034/j.1399-0039.2001.057002110.x

[22] Prigione I, Corrias MV, Airoldi I, Raffaghello L, Morandi F, et al (2004) Immunogenicity of human neuroblastoma. Ann N Y Acad Sci 1028: 69–80. 15650233 10.1196/annals.1322.008

[23] Raffaghello L, Prigione I, Bocca P, Morandi F, Camoriano M, et al (2005) Multiple defects of the antigen-processing machinery components in human neuroblastoma: immunotherapeutic implications. Oncogene 24: 4634–4644. 15897905 10.1038/sj.onc.1208594

[24] Neefjes J, Jongsma ML, Paul P, Bakke O (2011) Towards a systems understanding of MHC class I and MHC class II antigen presentation. Nat Rev Immunol 11: 823–836. 22076556 10.1038/nri3084

[25] Zhou F (2009) Molecular mechanisms of IFN-gamma to up-regulate MHC class I antigen processing and presentation. Int Rev Immunol 28: 239–260. 19811323 10.1080/08830180902978120

[26] Miller CH, Maher SG, Young HA (2009) Clinical Use of Interferon-gamma. Ann N Y Acad Sci 1182: 69–79. 20074276 10.1111/j.1749-6632.2009.05069.x PMC6574079

[27] Bracarda S, Eggermont AM, Samuelsson J (2010) Redefining the role of interferon in the treatment of malignant diseases. Eur J Cancer 46: 284–297. 19906524 10.1016/j.ejca.2009.10.013

[28] Lesinski GB, Zimmerer JM, Kreiner M, Trefry J, Bill MA, et al (2010) Modulation of SOCS protein expression influences the interferon responsiveness of human melanoma cells. BMC Cancer 10: 142. 20398276 10.1186/1471-2407-10-142 PMC2858748

[29] Motzer RJ, Escudier B, Oudard S, Hutson TE, Porta C, et al (2010) Phase 3 trial of everolimus for metastatic renal cell carcinoma : final results and analysis of prognostic factors. Cancer 116: 4256–4265. 20549832 10.1002/cncr.25219

[30] Drew PD, Franzoso G, Carlson LM, Biddison WE, Siebenlist U, et al (1995) Interferon regulatory factor-2 physically interacts with NF-kappa B in vitro and inhibits NF-kappa B induction of major histocompatibility class I and beta 2-microglobulin gene expression in transfected human neuroblastoma cells. J Neuroimmunol 63: 157–162. 8550813 10.1016/0165-5728(95)00140-9

[31] Drew PD, Franzoso G, Becker KG, Bours V, Carlson LM, et al (1995) NF kappa B and interferon regulatory factor 1 physically interact and synergistically induce major histocompatibility class I gene expression. J Interferon Cytokine Res 15: 1037–1045. 8746784 10.1089/jir.1995.15.1037

[32] Naumann M, Scheidereit C (1994) Activation of NF-kappa B in vivo is regulated by multiple phosphorylations. EMBO J 13: 4597–4607. 7925300 10.1002/j.1460-2075.1994.tb06781.x PMC395392

[33] Gobin SJ, Keijsers V, van Zutphen M, van den Elsen PJ (1998) The role of enhancer A in the locus-specific transactivation of classical and nonclassical HLA class I genes by nuclear factor kappa B. J Immunol. 161: 2276–2283. 9725221

[34] Gobin SJ, van Zutphen M, Woltman AM, van den Elsen PJ (1999) Transactivation of classical and nonclassical HLA class I genes through the IFN-stimulated response element. J Immunol 163: 1428–1434. 10415043

[35] Gobin SJ, Biesta P, Van den Elsen PJ (2003) Regulation of human beta 2-microglobulin transactivation in hematopoietic cells. Blood 101: 3058–3064. 12480693 10.1182/blood-2002-09-2924

[36] Moschonas A, Kouraki M, Knox PG, Thymiakou E, Kardassis D, et al (2008) CD40 induces antigen transporter and immunoproteasome gene expression in carcinomas via the coordinated action of NF-kappaB and of NF-kappaB-mediated de novo synthesis of IRF-1. Mol Cell Biol 28: 6208–6222. 18694960 10.1128/MCB.00611-08 PMC2577429

[37] Tanioka T, Hattori A, Mizutani S, Tsujimoto M (2005) Regulation of the human leukocyte-derived arginine aminopeptidase/endoplasmic reticulum-aminopeptidase 2 gene by interferon-gamma. FEBS J 272: 916–928. 15691326 10.1111/j.1742-4658.2004.04521.x

[38] Forloni M, Albini S, Limongi MZ, Cifaldi L, Boldrini R, et al (2010) NF-kappaB, and not MYCN, regulates MHC class I and endoplasmic reticulum aminopeptidases in human neuroblastoma cells. Cancer Res 70: 916–924. 20103633 10.1158/0008-5472.CAN-09-2582

[39] Doody GM, Stephenson S, McManamy C, Tooze RM (2007) PRDM1/BLIMP-1 modulates IFN-gamma-dependent control of the MHC class I antigen-processing and peptide-loading pathway. J Immunol 179: 7614–7623. 18025207 10.4049/jimmunol.179.11.7614

[40] Sigalotti L, Covre A, Zabierowski S, Himes B, Colizzi F, et al (2008) Cancer testis antigens in human melanoma stem cells: expression, distribution, and methylation status. J Cell Physiol 215: 287–291. 18205182 10.1002/jcp.21380 PMC3624762

[41] Giorda E, Sibilio L, Martayan A, Moretti S, Venturo I, et al (2003) The antigen processing machinery of class I human leukocyte antigens: linked patterns of gene expression in neoplastic cells. Cancer Res 63: 4119–4127. 12874016

[42] Saveanu L, Carroll O, Lindo V, Del Val M, Lopez D, et al (2005) Concerted peptide trimming by human ERAP1 and ERAP2 aminopeptidase complexes in the endoplasmic reticulum. Nat Immunol 6: 689–697. 15908954 10.1038/ni1208

[43] Martayan A, Fraioli R, Giorda E, Setini A, Ciccarelli G, et al (1999) Biosynthesis of HLA-C heavy chains in melanoma cells with multiple defects in the expression of HLA-A, -B, -C molecules. Br J Cancer 80: 639–649. 10360639 10.1038/sj.bjc.6690405 PMC2362293

[44] van Endert PM, Tampe R, Meyer TH, Tisch R, Bach JF, et al (1994) A sequential model for peptide binding and transport by the transporters associated with antigen processing. Immunity 1: 491–500. 7895159 10.1016/1074-7613(94)90091-4

[45] Stam NJ, Spits H, Ploegh HL (1986) Monoclonal antibodies raised against denatured HLA-B locus heavy chains permit biochemical characterization of certain HLA-C locus products. J Immunol 137: 2299–2306. 3760563

[46] Shimada H, Ambros IM, Dehner LP, Hata J, Joshi VV, et al (1999) The International Neuroblastoma Pathology Classification (the Shimada system). Cancer 86: 364–372. 10421273

[47] Gaugler B, Van den Eynde B, van der Bruggen P, Romero P, Gaforio JJ, et al (1994) Human gene MAGE-3 codes for an antigen recognized on a melanoma by autologous cytolytic T lymphocytes. J Exp Med 179: 921–930. 8113684 10.1084/jem.179.3.921 PMC2191409

[48] Fontana R, Bregni M, Cipponi A, Raccosta L, Rainelli C, et al (2009) Peripheral blood lymphocytes genetically modified to express the self/tumor antigen MAGE-A3 induce antitumor immune responses in cancer patients. Blood 113: 1651–1660. 19074732 10.1182/blood-2008-07-168666

[49] Russo V, Tanzarella S, Dalerba P, Rigatti D, Rovere P, et al (2000) Dendritic cells acquire the MAGE-3 human tumor antigen from apoptotic cells and induce a class I-restricted T cell response. Proc Natl Acad Sci U S A 97: 2185–2190. 10681453 10.1073/pnas.040540197 PMC15775

[50] Cifaldi L, Lo Monaco E, Forloni M, Giorda E, Lorenzi S, et al (2011) Natural killer cells efficiently reject lymphoma silenced for the endoplasmic reticulum aminopeptidase associated with antigen processing. Cancer Res 71: 1597–1606. 21252114 10.1158/0008-5472.CAN-10-3326

[51] Fruci D, Ferracuti S, Limongi MZ, Cunsolo V, Giorda E, et al (2006) Expression of endoplasmic reticulum aminopeptidases in EBV-B cell lines from healthy donors and in leukemia/lymphoma, carcinoma, and melanoma cell lines. J Immunol 176: 4869–4879. 16585582 10.4049/jimmunol.176.8.4869

[52] Fruci D, Giacomini P, Nicotra MR, Forloni M, Fraioli R, et al (2008) Altered expression of endoplasmic reticulum aminopeptidases ERAP1 and ERAP2 in transformed non-lymphoid human tissues. J Cell Physiol 216: 742–749. 18393273 10.1002/jcp.21454

[53] Santhekadur PK, Gredler R, Chen D, Siddiq A, Shen XN, et al (2012) Late SV40 Factor (LSF) Enhances Angiogenesis by Transcriptionally Up-regulating Matrix Metalloproteinase-9 (MMP-9). J Biol Chem 287: 3425–3432. 22167195 10.1074/jbc.M111.298976 PMC3270996

