# Supplementary figures and images for: IRF1 and NF-kB Restore MHC Class I-Restricted Tumor Antigen Processing and Presentation to Cytotoxic T Cells in Aggressive Neuroblastoma
Source: PLoS One. 2012 Oct 5;7(10):e46928. doi: 10.1371/journal.pone.0046928 (PMC3465322; doi:10.1371/journal.pone.0046928)

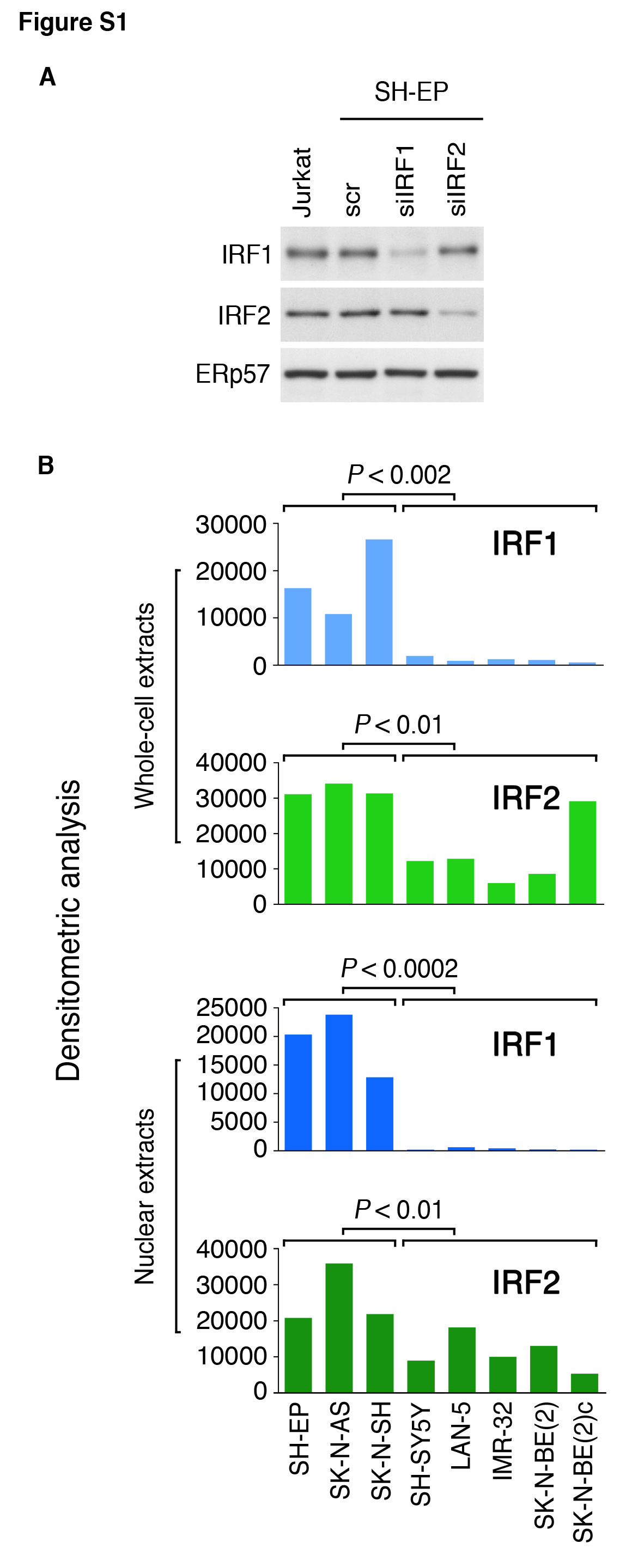

Supplement: Figure S1 — Specificity, densitometric and statistical analyses of IRF1 and IRF2 expression. (A) Immunoblot analysis of IRF1 and IRF2 in SH-EP cells transfected with either IRF1 siRNA (siIRF1), IRF2 siRNA (siIRF2) or control scrambles (scr). The Jurkat cell line was included as a positive control. Equal amounts of whole-cell extracts were resolved by SDS-PAGE, immunoblotted and probed with IRF1 and IRF2 antibodies. ERp57 was used for normalization. Silencing of IRF1 and IRF2 by RNA interference in SH-EP cells affects the levels of IRF1 and IRF2 polypeptides as compared with the controls demonstrating the specificity of the antibodies. (B) Densitometric and statistical analyses of the Western blot bands shown in Figure 1B. Western blot bands in Fig. 1B were submitted to densitometric analysis. IRF1 and IRF2 values of the 3 MHC-I-expressing NB cells (to the left) and the 5 MHC-I-low NB cells (to the right) were separately averaged, and statistical significance (P value) of the differences between the two series was calculated as described in Materials and Methods. (TIF) [file pone.0046928.s001.tif]

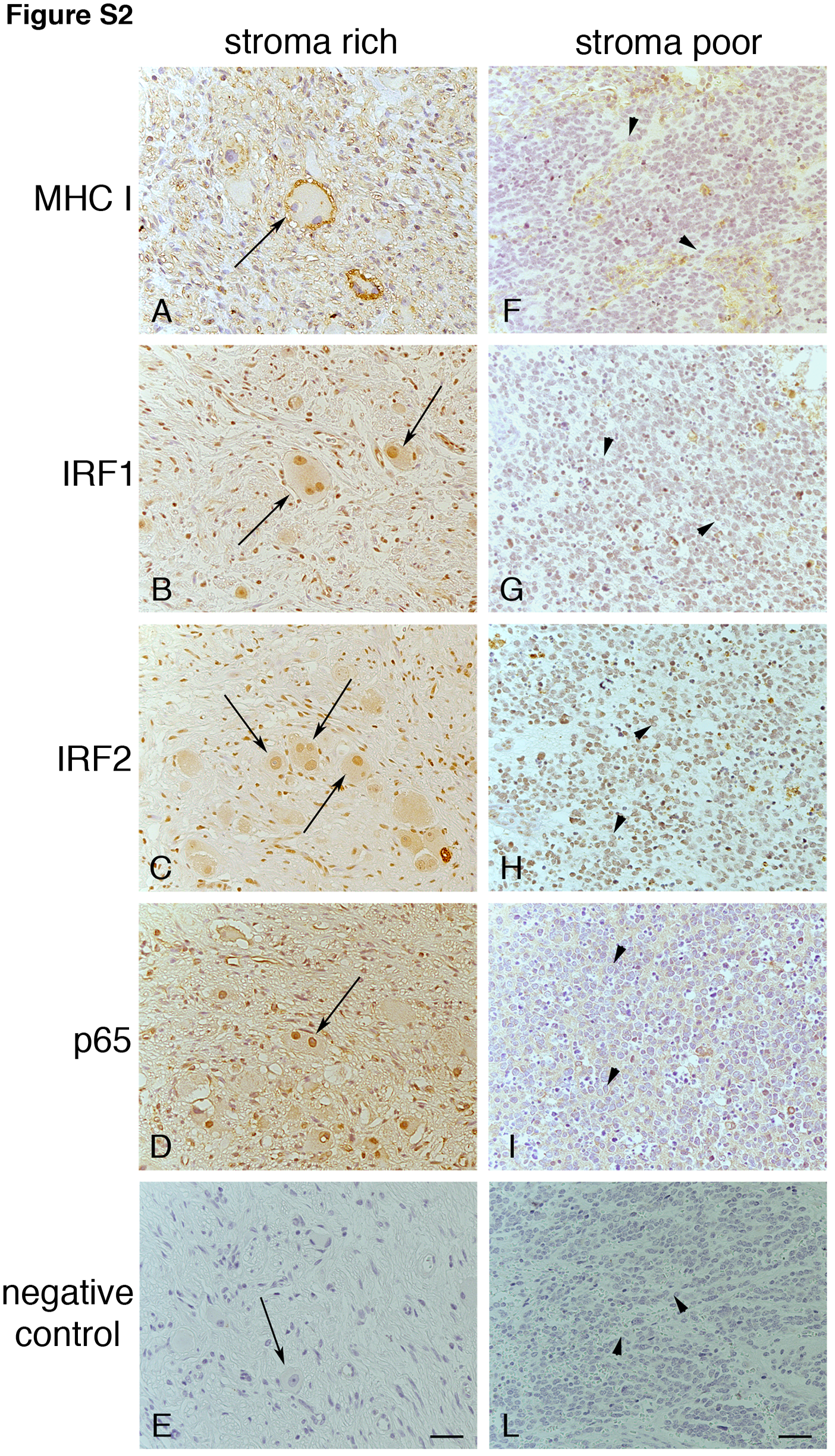

Supplement: Figure S2 — Immunohistochemistry of human NB tissue sections with antibodies to MHC-I (A, F), IRF1 (B, G), IRF2 (C, H), p65 (D, L) or negative controls (E, L) at original magnification, x20. Scale bars 60 µm. Expression of IRF1, IRF2 and p65 in the nuclei of mature ganglion cells (arrows), endothelial cells, lymphocytes and stroma cells in the MHC-I-positive ganglioneuroblastoma is shown in A to D. Weak expression of IRF1, IRF2 and p65 in the MHC-I-negative neuroblastic cells (arrowhead), is shown in F to I. Data shown are representative of 10 stroma-rich and 10 stroma-poor NB tissue sections. (TIF) [file pone.0046928.s002.tif]

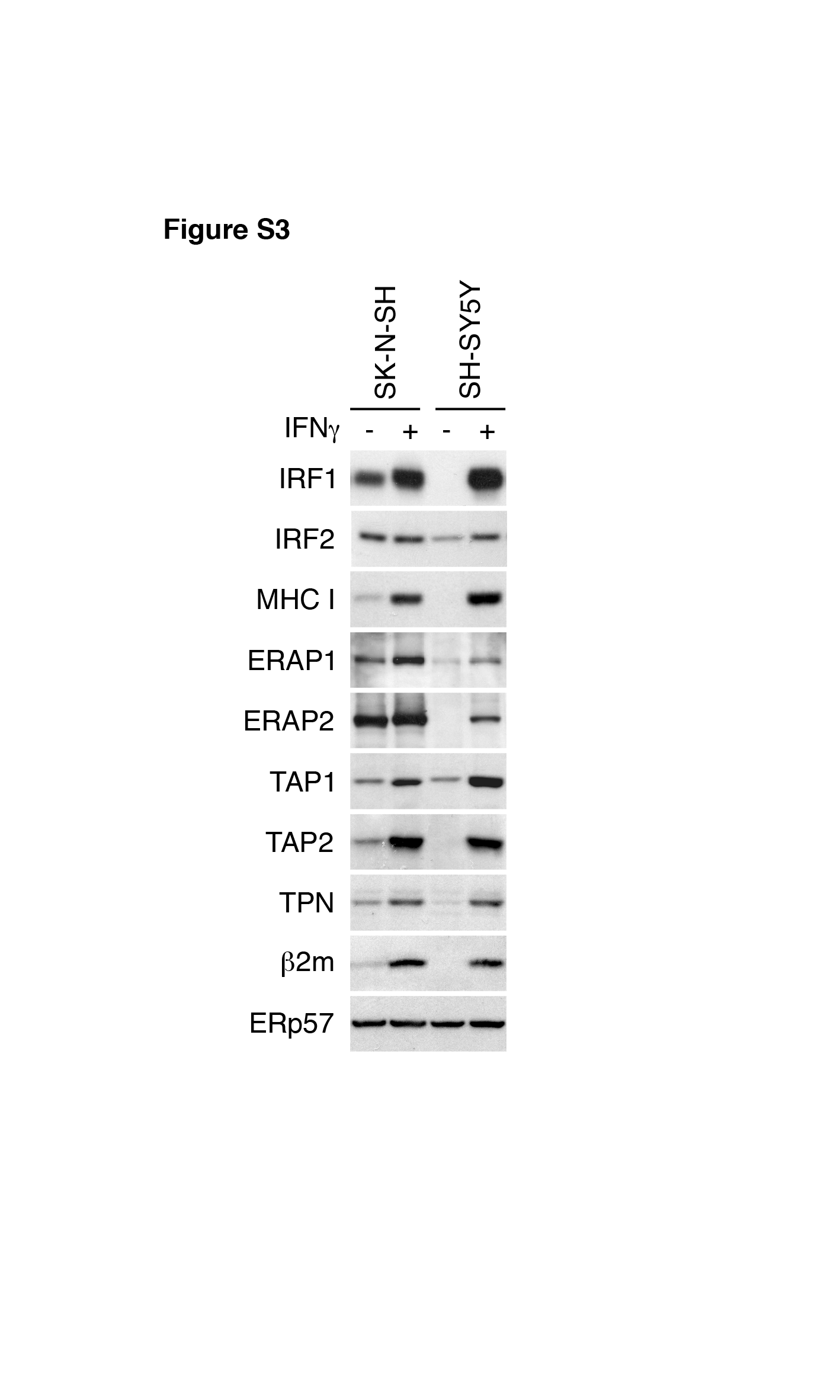

Supplement: Figure S3 — Lysates of SK-N-SH and SH-SY-5Y cell lines cultured for 48 hours in the presence and absence of IFN-γ were resolved by SDS-PAGE and immunoblotted and probed with the indicated antibodies. ERp57 was used for normalization. Data are representative of 3 independent experiments. (TIF) [file pone.0046928.s003.tif]

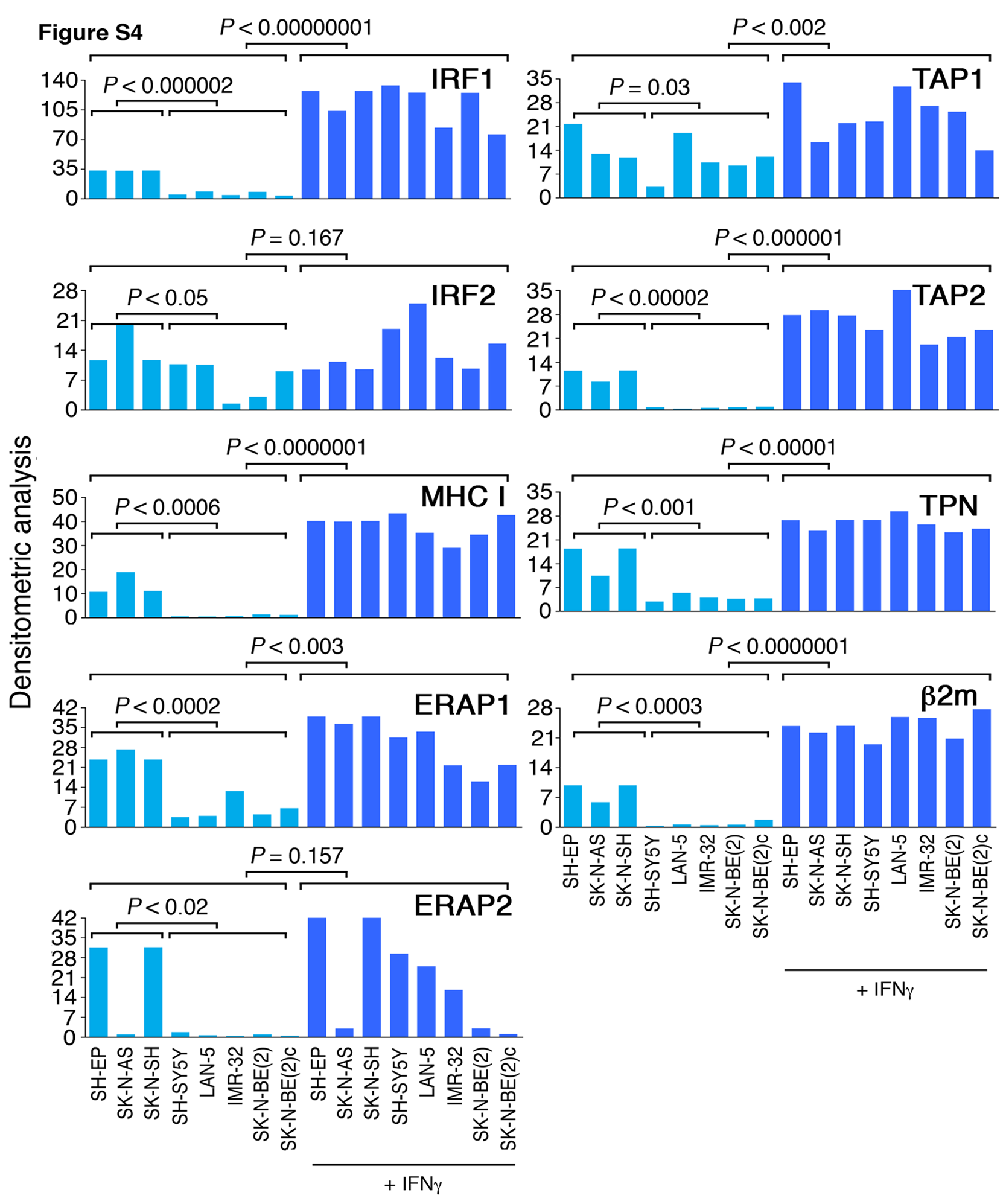

Supplement: Figure S4 — Densitometric and statistical analyses of the immunoblots shown in Figure 3A . Densitometric values of the IFN-γ-treated (+IFNγ) or left untreated NB cells were separately averaged, and statistical significance (P value) of the differences between the two series was calculated as described in Materials and Methods. In addition, densitometric values of the 3 MHC-I-expressing NB cells and the 5 untreated MHC-I-low NB cells both untreated were separately averaged, and statistical significance (P value) of the differences between the two series was calculated as described above. (TIF) [file pone.0046928.s004.tif]

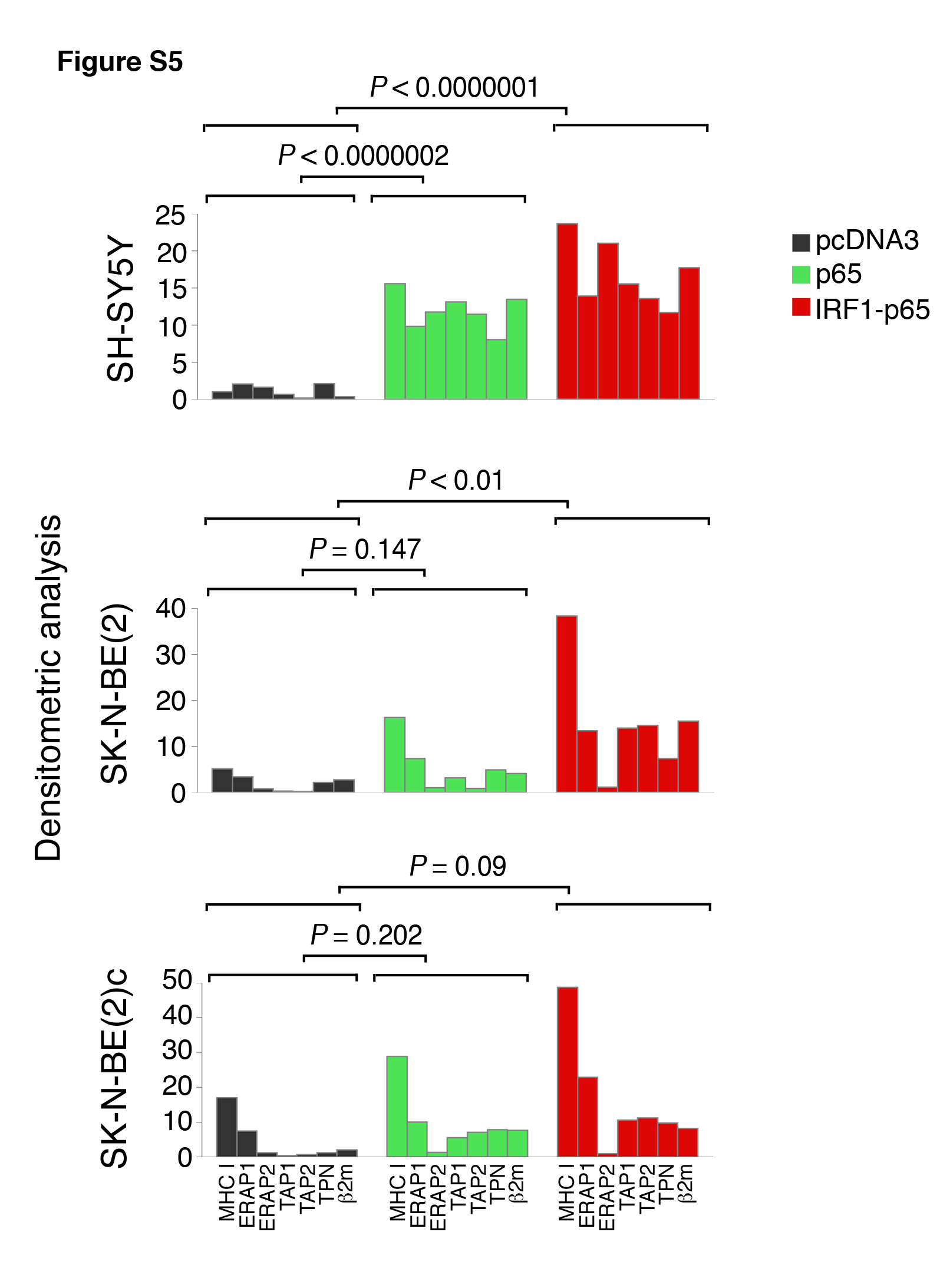

Supplement: Figure S5 — Densitometric and statistical analyses of the immunoblots shown in Figure 4A . Western blot bands were submitted to densitometric analysis. Densitometric values of cells transfected with pcDNA3, NF-kB p65 subunit, or IRF1 and NF-kB p65 subunit were separately averaged, and statistical significance (P value) of the differences between the three series was calculated as described in Materials and Methods. (TIF) [file pone.0046928.s005.tif]

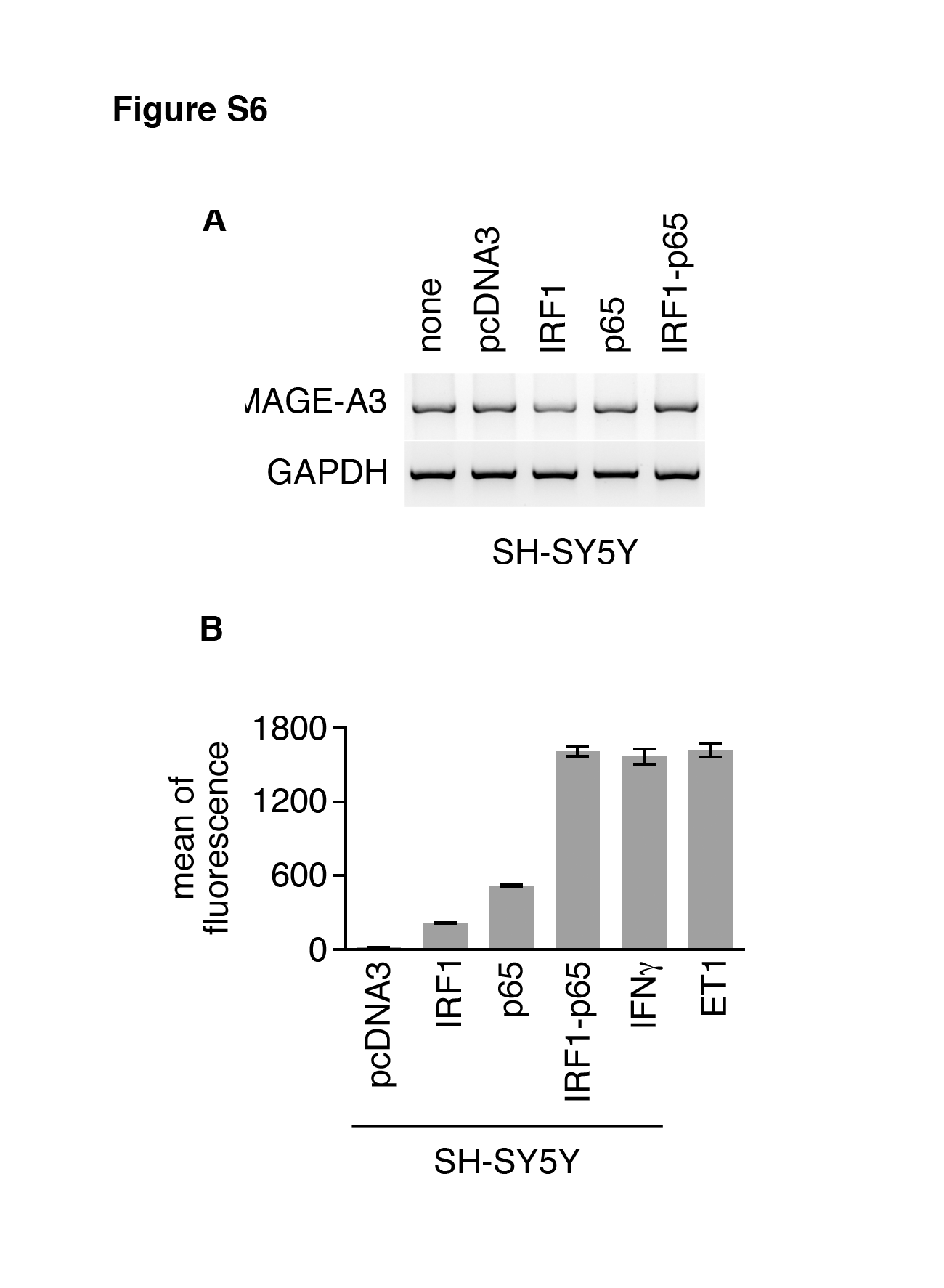

Supplement: Figure S6 — A, RT-PCR analysis of MAGE-A3 in the SH-SY5Y either untransfected (none) and transfected with IRF1 and/or the NF-kB p65 subunit and the control empty vector (pcDNA3). Total mRNA was extracted from the transfected cells, reverse transcribed and cDNAs amplified with specific primers for MAGE-A3. GAPDH gene was used for normalization. B, flow cytometry analysis of surface MHC-I expression of SH-SY5Y cells treated as described in Figure 5 using W6/32 mAb. Data are shown as mean of fluorescence. (TIF) [file pone.0046928.s006.tif]
